# Supplementary material for: Resolving the full spectrum of human genome variation using Linked-Reads
Source: Genome Res. 2019 Apr;29(4):635–45. doi: 10.1101/gr.234443.118 (PMC6442396; doi:10.1101/gr.234443.118)
Supplement: Supplemental Material [file supp_gr.234443.118_Supplemental_longranger-2.2.zip › longranger-2.2.tar.bz2!/lr2.2-public/lib/go/src/code.google.com/vitess/docs/user-guide/update-stream.html]

Vitess / Update Stream


Toggle navigation

- Overview
- Guides
- Reference
- Blog
- GitHub

- #### Overview

  - What is Vitess
  - Scaling MySQL with Vitess
  - Key Concepts
- #### Getting Started

  - Run Vitess on Kubernetes
    - Custom Docker Build
  - Run Vitess Locally
- #### User Guide

  - Introduction- Client Libraries- Backing Up Data- Reparenting
        - Schema Management
        - VSchema Guide
        - Schema Swap (Tutorial)
        - Sharding
          - Horizontal Sharding (Tutorial, automated)
          - Horizontal Sharding (Tutorial, manual)
          - Sharding in Kubernetes (Tutorial, automated)
          - Sharding in Kubernetes (Tutorial, manual)
        - Vitess Sequences
        - MySQL Server Protocol
        - Vitess and Replication
        - Update Stream
        - Row Based Replication
        - Topology Service
        - Transport Security Model
        - Launching
          - Scalability Philosophy
          - Production Planning
          - Server Configuration
          - 2PC Guide
          - Troubleshooting
        - Upgrading
- #### Reference Guides

  - Vitess API- vtctl Commands
- #### Other Resources

  - Presentations- Blog- Roadmap
- #### Contributing

  - Contributing to Vitess- GitHub Workflow- Code Reviews
- GitHub

# Update Stream

- #### Overview

  - What is Vitess
  - Scaling MySQL with Vitess
  - Key Concepts
- #### Getting Started

  - Run Vitess on Kubernetes
    - Custom Docker Build
  - Run Vitess Locally
- #### User Guide

  - Introduction- Client Libraries- Backing Up Data- Reparenting
        - Schema Management
        - VSchema Guide
        - Schema Swap (Tutorial)
        - Sharding
          - Horizontal Sharding (Tutorial, automated)
          - Horizontal Sharding (Tutorial, manual)
          - Sharding in Kubernetes (Tutorial, automated)
          - Sharding in Kubernetes (Tutorial, manual)
        - Vitess Sequences
        - MySQL Server Protocol
        - Vitess and Replication
        - Update Stream
        - Row Based Replication
        - Topology Service
        - Transport Security Model
        - Launching
          - Scalability Philosophy
          - Production Planning
          - Server Configuration
          - 2PC Guide
          - Troubleshooting
        - Upgrading
- #### Reference Guides

  - Vitess API- vtctl Commands
- #### Other Resources

  - Presentations- Blog- Roadmap
- #### Contributing

  - Contributing to Vitess- GitHub Workflow- Code Reviews

# Update Stream

Update Stream is a Vitess service that provides a change stream for any keyspace.
The use cases for this service include:

- Providing an *invalidation stream*, that an application can use to maintain a cache.
- Maintain an external copy of the data in another system, that is only updated
  when the data changes.
- Maintain a change record of all the transactions that have been applied to the data.

A good understanding
of Vitess Replication is required to
understand this document better. We will go through the use cases in a bit more
details, then introduce the EventToken notion, and finally explain the service.

## Use Cases

### Maintaining Cache Consistency

The first use case we’re trying to address is to maintain a consistent cache of
the data. The problem here has two parts:

- When data changes, we need to invalidate the cache.
- When we want to re-populate the cache after an invalidation, we need to make
  sure we get data that is more recent than the data change. For instance, we
  can’t just re-query any replica, as it might be behind on replication.

This process can be somewhat resilient to some stream anomalies. For instance,
invalidating the same record twice in some corner cases is fine, as long as we
don’t poison the cache with an old value.

Note the location / ownership of the cache is not set in stone:

- Application-layer cache: the app servers maintain the cache. It’s very early
  in the serving chain, so in case of a cache hit, it’s lower latency. However,
  an invalidation process needs to run and is probably also owned by the
  application layer, which is somewhat annoying.
- vtgate-layer cache: it would be a row cache accessed by vtgate, transparent to
  the app. It requires vtgate to do a lot of extra heavy-lifting, depending on
  what we want to support. Cache invalidation is still required, at a row level.
- vttablet-layer cache: this is the old rowcache. Since the cache is not shared
  across instances, and the app still needs a cache, we abandoned this one.

Since the vtgate-layer cache is much harder to work on (because of the query
implications), and will probably require similar components as the app-layer
cache, we decided to work on the app-layer cache for now, with possibly an
invalidation process that is somewhat tied to the app.

The *composite object cache* is an interesting use case: if the application is
in charge of the cache, it would seem possible to put in the cache higher level
composite objects, that are built from multiple table records. They would be
invalidated any time one of the composing table record is changed. They need to
be addressed by a part of the primary key, so they’re easy to find.

### Change Log

A Change Log provides a stream of all data changes, so an external application
can either record these changes, or keep an external database up to date with
the latest data.

Unlike the Cache Invalidation use case, this is not as forgiving. If we have
duplicate updates, they will need to be handled.

## Design Considerations

### Single Shard Update Stream

This has been supported in Vitess for a while, but not exposed. It works as follows:

- vttablet adds an SQL comment to every DML, that contains the Primary Key of
  the modified row.
- vttablet provides a streaming service that can connect to the local MySQL
  replication stream, extract the comment, and stream events to the client.
- Use the GTID as the start / restart position. It is sent back with each
  update, and an Update Stream can be started from it.

Note Vitess supports both the MariaDB GTIDs (domain:server:sequence) and the
MySQL 5.6 GTID Sets (encoded in SID blocks).

### Surviving Resharding: Problem

The Vitess tools are supposed to provide transparent sharding for the user’s
data. Most of the trouble we run into is surviving resharding events, when we
hop over from one set of shards to another set of shards.

Two strategies then come to mind:

- Provide a per-shard update stream. Let the user handle the hop when resharding
  happens. If we were to do this for the Cache use case, we would also need to
  provide some way of preventing bad corner cases, like a full cache flush, or
  no cache update for a while, or lost cache invalidations. Simple for us, but
  the app can be a lot more complicated. And the Change Log use case is also
  hard to do.
- Provide a per-keyrange update stream. Vtgate would connect to the right
  shards, and resolve all conflicts. We can add the restriction that the client
  only asks for keyranges that are exactly matching to one or more shards. For
  instance, if a keyspace is sharded four ways, -40, 40-80, 80-c0, c0-, we can
  support clients asking for -40, -80, -, but not for 60-a0 for instance.

As a reminder, the resharding process is somewhat simple:

- Let’s say we want to split a shard 20-40 into two shards, 20-30 and 30-40. At
  first, only 20-40 exists and has a GTID stream.
- We create 20-30 and 30-40, each has its own GTID stream. We copy the schema,
  and the data.
- Filtered replication is enabled. A transaction in 20-40 is replayed on both
  20-30 and 30-40, with an extra blp\_checkpoint statement, that saves the 20-40
  GTID.
- At some point, we migrate the read-only traffic from 20-40 replicas to 20-30
  and 30-40 replicas. (Note: this is probably when we want to migrate any
  invalidation process as well).
- Then as a final step, the writes are migrated from 20-40 to 20-30 and 30-40.

So we have a window of time when both streams are available simultaneously. For
the resharding process to be operationally better, that window should be as
small as possible (so we don't run with two copies of the data for too long). So
we will make sure an Update Stream can hop from the source shards to the
destination shards quickly.

### Surviving Resharding: First Try

To solve the shard hop problem during resharding, we tried to explore adding
good timing information to the replication stream. However:

- Since the time is added by vttablet, not MySQL, it is not accurate, not
  monotonic, and provides no guarantees.
- Which such loose guarantees, it is no better than the second-accurate
  timestamp added by MySQL to each transaction.

So this idea was abandoned.

The GTID stream maintained by MySQL is the only true source of IDs for
changes. It’s the only one we can trivially seek on, and get binlogs. The main
issue with it is that it’s not maintained across shards when resharding.

However, it is worth noting that a transaction replicated by the binlog streamer
using Filtered Replication also saves the original GTID and the source
transaction timestamp in the blp\_checkpoint table. So we could extract the
original GTID and timestamp from at least that statement (and if not, from an
added comment).

### Change Log and SBR

If all we have is Statement Based Replication (SBR), we cannot get an accurate
Change Log. SBR only provides the SQL statements, there is no easy way for us to
parse them to get the final values of the columns (OK, there is, it’s just too
complicated). And we cannot just query MySQL, as it may have already applied
more transactions related to that record. So for Change Log, we need Row Based
Replication (or a more advanced replication system).

Note we can use the following setup:

- Master and replicas use SBR.
- Rdonly use SBR to connect to master, but log RBR logs locally.
- We get the replication stream from rdonly servers.

This is a bit awkward, and the main question is: what happens if a rdonly server
is the only server that has replicated and semi-sync-acked a transaction, while
the master is dying? Then to get that change, the other servers would get the
RBR version of the change.

Vitess support for RBR is coming. We will then explore these use cases further.

## Detailed Design

In the rest of this document, we’ll explore using the GTID when tracking a
single shard, and revert to the timestamp when we hop across shards.

As we do not want the application layer to understand / parse / compare the
GTIDs, we’ll use an opaque token, and just pass it around the various
layers. Vtgate / vttablet will understand it. The invalidation process should
not have to, but will as there is no better solution.

This approach can be made to work for the cache invalidation use case, but it
might be difficult to provide an exact point in time for recovery / switching
over to a different set of shards during resharding.

For the Change Log, we’ll see what we can provide.

### Event Token

We define an Event Token structure that contains:

- a MySQL replication timestamp (int64, seconds since Epoch).
- a shard name
- A GTIDSet position.

It basically describes a position in a replication stream.

An Event Token is always constructed from reading a transaction from the
binlogs. If filtered replication is running, we use the source shard timestamp.

Event Token comparison:

- First, if the timestamps are different, just use that.
- Then, if both use the same shard name, compare the GTIDs.
- Otherwise we do not know for sure. It will depend on the usage to figure out
  what we do.

*Possible Extension*: when filtered replication is running, we also update
blp\_checkpoint with the source GTID. We could add that information to the Event
Token. Let’s try to go without in the first version, to remain simple. More on
this later in the ‘Data Dump, Keeping it up to Date’ section.

### Vttablet Changes

#### Watching the Replication Stream

Replicas are changed to add a background routine that reads the binlogs
(controlled by the `watch_replication_stream` flag). When a tablet’s type is set
to `replica`, the routine starts. It stops when the tablet is not `replica` any
more (goes to `master`, `worker`, …).

The routine starts reading the binlog from the current position. It then remembers:

- The Event Token of the last seen transaction.
- *Possible Optimization*: A map of the first time a timestamp is seen to the
  corresponding GTID position and filename / position. This would be a value per
  second. Let’s age these out: we keep the values for the last N seconds, then
  we keep a value for every minute for the last M hours. We forget values older
  than 3 days (or whatever the binlog retention time is).

#### `include_event_token` Option

We added an option to the Query Service API for Execute calls, called
`include_event_token`. If set, vttablet will get the last seen Event Token right
before issuing the query to MySQL, and include it in the response. This
essentially represents the last known replication position that we’re sure the
data we’re returning is fresher than.

#### `compare_event_token` Option

We added an option to the Query Service API for Execute calls, called
`compare_event_token`. The provided event token is sent along with the call, and
vttablet compares that token with the one its current replication stream watcher
has. It returns the result of the comparison in ResultExtras.

#### Update Stream API Change

The Update Stream API in vttablet right now can only start from a GTID. We added a
new API that can start from a timestamp as well. It will look for the right
binlog file to start with, and start streaming events, discarding events until
it finds the provided timestamp. *Optimization*: It can also look in the map to
find the closest value it can start with, and then read from the binlogs until
it finds the first timestamp. If it doesn’t have old enough values in its map,
it errors out (the goal is to have vtgate then try another tablet to start
from). For each event, we will also return the corresponding Event Token.

*Optimization*: if an Update Stream client is caught up to the current binlog
reading thread, we can just tee the binlog stream to that client. We won’t do
that in the first version, as we don’t expect that many clients.

Note that when filtered replication is running, we need to have the timestamp of
the source transaction on the source shard, not the local timestamp of the
applied transaction. Which also means that timestamps will not be always
linearly increasing in the stream, in the case of a shard merge (although they
will be linearly increasing for a given keyspace\_id).

### Vtgate Changes

We added a new Update Stream service to vtgate. It takes as input a keyspace and
an optional KeyRange (for sharded keyspaces). As a starting point, it takes a
timestamp.

*Caveat*: As previously mentioned, at first, we can add the restriction that the
client only asks for KeyRanges that are exactly matching to one or more
shards. For instance, if a keyspace is sharded four ways, -40, 40-80, 80-c0,
c0-, we can support clients asking for -40, -80, -, but not for 60-a0 for
instance. Lifting that restriction is somewhat easy, we’d just have to filter
the returned keyspace\_ids by KeyRange, but that’s extra work for not much gain
in the short term (and we don’t parse keyspace\_id in Binlog Streamer right now,
just the PK).

After using the partition map in SrvKeyspace, vtgate will have a list of shards
to query. It will need to create a connection for every shard that overlaps with
the input KeyRange. For every shard, it will pick an up-to-date replica and use
the Update Stream API mentioned above. If the vttablet cannot provide the
stream, it will failover to another one. It will then start an Update Stream on
all sources, and just merge and stream the results back to the source. For each
Event Token that is read from a source, vtgate will also send the smallest
timestamp of all Events it’s seen in all sources. That way the client has a
value to start back from in case it needs to restart.

In case of resharding event, the list of shards to connect to may change. Vtgate
will build a map of overlapping shards, to know which source shards are mapped
to which destination shards. It will then stop reading from all the source
shards, find the minimum timestamp of the last event it got from each source,
and use that to restart the stream on the destination shards.

*Alternate Simpler Solution*: when vtgate notices a SrvKeyspace change in the
serving shards, it just aborts the invalidation stream. The client is
responsible for reconnecting with the last timestamp it’s seen. The client will
need to handle this error case anyway (when vtgates get upgraded at least).

*Caveat*: this will produce duplicate Event Tokens, with the same timestamp but
with GTID positions from two different streams. More on this later, but for a
Cache Invalidation scenario, no issue, and for a Change Log application, we’ll
see how we can deal with it.

We also add the same `include_event_token` flag to vtgate query service. It just
passes it along to the underlying vttablet. It’s only supported for
single-keyspace\_id queries. The resulting EventToken is just returned back as
is.

## Use Cases How To

Let's revisit our use cases and see how this addresses them.

### Cache Invalidation

The idea is to use the Event Token coming from both the Execute results and the
Update Stream to maintain cache consistency.

The cache contains entries with both:

- An Event Token. It describes either the invalidation, or the last population.
- An optional value.

The invalidation process works as follows:

- It asks vtgate for an Update Stream for a provided keyspace / KeyRange,
  starting at the current timestamp (or from a few seconds/minutes/hours in the
  past, or from the last checkpointed timestamp it had saved).
- Vtgate resolves the keyrange into shards. It starts an invalidation stream
  with a healthy replica in each shard from the provided timestamp.
- Vtgate sends back all Event Tokens it collects, with all of timestamp, shard
  name and GTID.
- For each change it gets, the invalidation process reads the cache record. Two cases:

  - No entry in the cache: it stores the Event Token (to indicate the cache
    should not be populated unless the value is greater) with no value.
  - An entry in the cache exists, with an Event Token:
  - If the cached Event Token is strictly older, update it with the new Event
    Token, clear the value.
  - If the cached Event Token is strictly more recent, discard the new Event.
  - If we don’t know which Event Token is the most recent (meaning they have
    the same timestamp, and are read from different invalidation stream), we
    need to do the safest thing: invalidate the cache with the current Event
    Token. This is the safest because we’re guaranteed to get duplicate
    events, and not miss events.
  - In any case the invalidation process only updates the cache if it still
    contains the value it read (CAS). Otherwise it rereads and tries again
    (means an appserver or another invalidator somehow also updated the cache).

A regular appserver will query the cache for the value it wants. It will get either:

- No entry: asks vtgate for the Event Token when querying the database, use a
  CAS operation to set the value the returned Event Token + Value.
- An entry with both an Event Token and a Value: Just use the value.
- An entry with just an Event Token and no Value:

  - Send the Event Token along with the query to vtgate as
    `compare_event_token`, and also asking for Event Token using `include_event_token`.
  - Vtgate will query vttablet as usual, but also passing both flags.
  - Vttablet will then compare the provided Event Token with the one that was
    included. It will include in the response the knowledge of the Event Token
    comparison as a boolean, only set if the data read is `fresher`.
  - Depending on the `fresher` boolean flag, the app will:
  - Data read is more recent: Update the cache with new Event Token / Value.
  - Data read is not more recent (or we don't know for sure): don’t update the cache.

*Constraints*:

- When restarting the invalidation process, we start from a point back in time,
  let’s say N seconds behind now. Since we can ask destination shards at this
  point for events that are N seconds old, filtered replication has to have been
  running for at least N seconds. (Alternatively, the invalidators can
  checkpoint their current position from time to time, and restart from that
  when starting up, and revert back to N seconds behind now).
- As mentioned before, the shard range queried by the invalidation process
  should cover a round number of actual shards.
- The invalidation process needs to know how to compare tokens. This is a
  bummer, I don’t see any way around it. We could simplify and only do the
  timestamp comparison part, but that would mean the cache is unused for up to
  an entire second upon changes. The appserver doesn’t need to compare, it gives
  the value to vtgate and let it do the work.

To see a sample use of the Update Stream feature, look at
the
cache\_invalidation.py integration
test. It shows how to do the invalidaiton in python, and the application
component.

### Extension: Removing Duplicate Events

In the previous section, we use timestamps to easily seek on replication
streams. If we added the ability to seek on any source GTID that appears in the
destination stream, we should be able to precisely seek at the right spot. That
would make exact transitions from one stream to the next possible. Again, as
long as the destination shard in a resharding event has been running filtered
replication for as long as we want to go back.

However, describing a position on a replication stream becomes tricky: it needs
one Event Token per replication stream. When resharding the Event Tokens would
jump around. When restarting a stream from an Event Token list, we may need to
restart earlier in some cases and skip some items.

*Bottom Line*:

- This would require a bunch of non-trivial code.
- This requires that filtered replication would be running for at least as long
  as we want to go back in time for the starting point.

If there is no use case for it, let’s not do it.

### Extension: Adding Update Data to the Stream, Towards Change Log

Let’s add a flag to the streaming query, that, if specified, asks for the
changed columns as well as the PK.

- If using SBR, and the flag is present, vttablet can just query the row at the
  time we get the event, and send it along. As already mentioned, the data may
  not be exactly up to date. It is however guaranteed to be newer than the Event
  Token, which might be good enough to put in a cache for instance.
- If using RBR, we just get the data for free, just send it along.

*Bottom Line*: Let’s try to go without this extension and see how it goes. We
can implement the additional data when we fully support RBR.

### Extension: Data Dump, Keeping It Up To Date

*Use Case*: keep a secondary database (like a HBase database) up to date.
*Requirements*: RBR replication, plus Data included in the Stream (previous extension).

It’s simple:

- The external database has the same schema as MySQL. Each row is indexed by
  PK. It also has an extra field, for the last Event Token.
- Remember start time of the process, let’s call it StartTime
- Dump the data to other database. Using Map/Reduce, whatever. Do not populate
  the Event Tokens.
- Start an invalidation process, asking for changes from StartTime. When getting
  updates, read the current external database row and its Event Token:

  - If there is no existing row / no Event token, save the new value.
  - If there is an existing row with a strictly more recent Event Token, ignore
    the event.
  - Otherwise (when the existing Event Token is older or we don’t know), store
    the new Value / Event Token.

Note this again means the dumping process needs to be able to compare Event
Tokens, as the invalidator does.

*Caveat*: As described, the values in the secondary storage will converge, but
they may go back in time for a bit, as we will process duplicate events during
resharding, and we may not know how to compare them.

*Extension*: if we also add the source GTID in Event Tokens read from a
destination shard during filtered replication, we can break the tie easily on
duplicate events, and guarantee we only move forward. This seems like the
easiest solution, and we can then use only timestamps as starting times for
restarting the sync process.

Contact: vitess@googlegroups.com  **·**  
Announcements  **·**  
© 2017 Vitess powered by Google Inc
